# KDM5A and PHF2 positively control expression of pro-metastatic genes repressed by EWS/Fli1, and promote growth and metastatic properties in Ewing sarcoma

**DOI:** 10.18632/oncotarget.27737

**Published:** 2020-10-27

**Authors:** Tyler S. McCann, Janet K. Parrish, Joseph Hsieh, Marybeth Sechler, Lays M. Sobral, Chelsea Self, Kenneth L. Jones, Andrew Goodspeed, James C. Costello, Paul Jedlicka

**Affiliations:** ^1^Department of Pathology, University of Colorado Denver, Anschutz Medical Campus, Aurora, CO, USA; ^2^Medical Scientist Training Program, University of Colorado Denver, Anschutz Medical Campus, Aurora, CO, USA; ^3^Cancer Biology Graduate Program, University of Colorado Denver, Anschutz Medical Campus, Aurora, CO, USA; ^4^Department of Pediatrics, University of Colorado Denver, Anschutz Medical Campus, Aurora, CO, USA; ^5^Department of Pharmacology, University of Colorado Denver, Anschutz Medical Campus, Aurora, CO, USA; ^6^Bioinformatics Shared Resource, University of Colorado Cancer Center, Anschutz Medical Campus, Aurora, CO, USA

**Keywords:** Ewing sarcoma, metastasis, epigenetics, Jumonji-domain histone demethylase

## Abstract

Ewing sarcoma is an aggressive malignant neoplasm with high propensity for metastasis and poor clinical outcomes. The EWS/Fli1 oncofusion is the disease driver in > 90% of cases, but presents a difficult therapeutic target. Moreover, EWS/Fli1 plays a complex role in disease progression, with inhibitory effects on critical steps of metastasis. Like many other pediatric cancers, Ewing sarcoma is a disease marked by epigenetic dysregulation. Epigenetic mechanisms present alternative targeting opportunities, but their contributions to Ewing sarcoma metastasis and disease progression remain poorly understood. Here, we show that the epigenetic regulators KDM5A and PHF2 promote growth and metastatic properties in Ewing sarcoma, and, strikingly, activate expression many pro-metastatic genes repressed by EWS/Fli1. These genes include L1CAM, which is associated with adverse outcomes in Ewing sarcoma, and promotes migratory and invasive properties. KDM5A and PHF2 retain their growth promoting effects in more metastatically potent EWS/Fli1^low^ cells, and PHF2 promotes both invasion and L1CAM expression in this cell population. Furthermore, KDM5A and PHF2 each contribute to the increased metastatic potency of EWS/Fli1^low^ cells *in vivo*. Together, these studies identify KDM5A and PHF2 as novel disease-promoting factors, and potential new targets, in Ewing sarcoma, including the more metastatically potent EWS/Fli1^low^ cell population.

## INTRODUCTION

Ewing sarcoma, the second most common cancer of bone and soft tissue in children and young adults, is a biologically and clinically aggressive malignancy, with strong tendency toward metastasis and poor long-term outcomes [1, 2]. Molecularly, Ewing sarcoma is a mutationally quiescent disease, characterized by expression of EWS/Ets, and, rarely other variant, fusion oncogenes that arise from specific, recurrent, chromosomal translocations [2–5]. These translocations yield in-frame fusion of the amino terminus of the EWS gene and the carboxy terminus, including the DNA-binding domain, of an Ets gene. EWS/Ets fusions, of which EWS/Fli1 is by far the most common (representing > 90% cases), exert multiple effects in the cell, acting as: pioneer factors to induce *de novo* enhancer elements; chromatin context-dependent transcriptional activators and repressors; and modulators of RNA processing [2–7]. Through these and other mechanisms, EWS/Fli1 and related fusions effect dramatic alterations in gene expression, which drive aberrant cell proliferation and survival, and are necessary for tumorigenesis.

While the role of EWS/Fli1 as driver of aberrant cell proliferation, survival, oncogenic transformation and tumor growth is well established, much less is known about the mechanisms underpinning the high metastatic propensity of Ewing sarcoma. Notably, while EWS/Fli1 imposes positive regulatory control over some pro-metastatic genes and pathways (eg: EZH2 [8] and PPP1R1A [9]), multiple studies indicate that, on balance, EWS/Fli1 exerts a repressive effect on important metastatic properties in Ewing sarcoma. Namely, EWS/Fli1: inhibits cell adhesion, motility and invasion *in vitro* [10, 11]; represses the expression of many metastasis-promoting genes [11–13]; and inhibits organ colonization and metastasis development in tail vein injection models *in vivo* [10, 11]. Thus, cells with low, rather than high, levels of EWS/Fli1 expression are more migratory, invasive and metastatically potent. Interestingly, recent studies demonstrate that EWS/Fli1 expression is quantitatively heterogeneous within both patient-derived cell lines and tumors, with some cells expressing high EWS/Fli1 levels, and some expressing low levels [11]. Together, the above observations suggest that “EWS/Fli1^low^” cells play an important role in the aggressive behavior of Ewing sarcoma.

Recent studies have increasingly demonstrated a critical role for epigenetic mechanisms in Ewing sarcoma pathogenesis. This includes identification of important roles played by the NuRD repressor complex and LSD1 [14], BMI1 [15], EZH2 [8], members of the BET bromodomain family [16–18], the chromatin remodeling BAF complex [6], and KDM3A (our prior studies [19–21]). As broad regulators of gene expression, like EWS/Fli1 itself, epigenetic modifiers have the potential to exert profound effects on disease phenotypes. Understanding of the biology of epigenetic modifiers thus has the potential to both inform important disease mechanisms, and, since many such modifiers are tractable therapeutic targets, identify possible new approaches to targeted therapy. Providing proof of concept, studies of BET inhibitors and LSD1 inhibitors have progressed to clinical trials in Ewing sarcoma. However, the biology of the vast majority of epigenetic modifiers, and their phenotypic and mechanistic intersections with the EWS/Fli1 driver oncofusion, remain to be defined in Ewing sarcoma.

## RESULTS

### The Jumonji-domain histone demethylases KDM5A and PHF2 are novel disease-promoting factors in Ewing sarcoma

**Figure 1:**
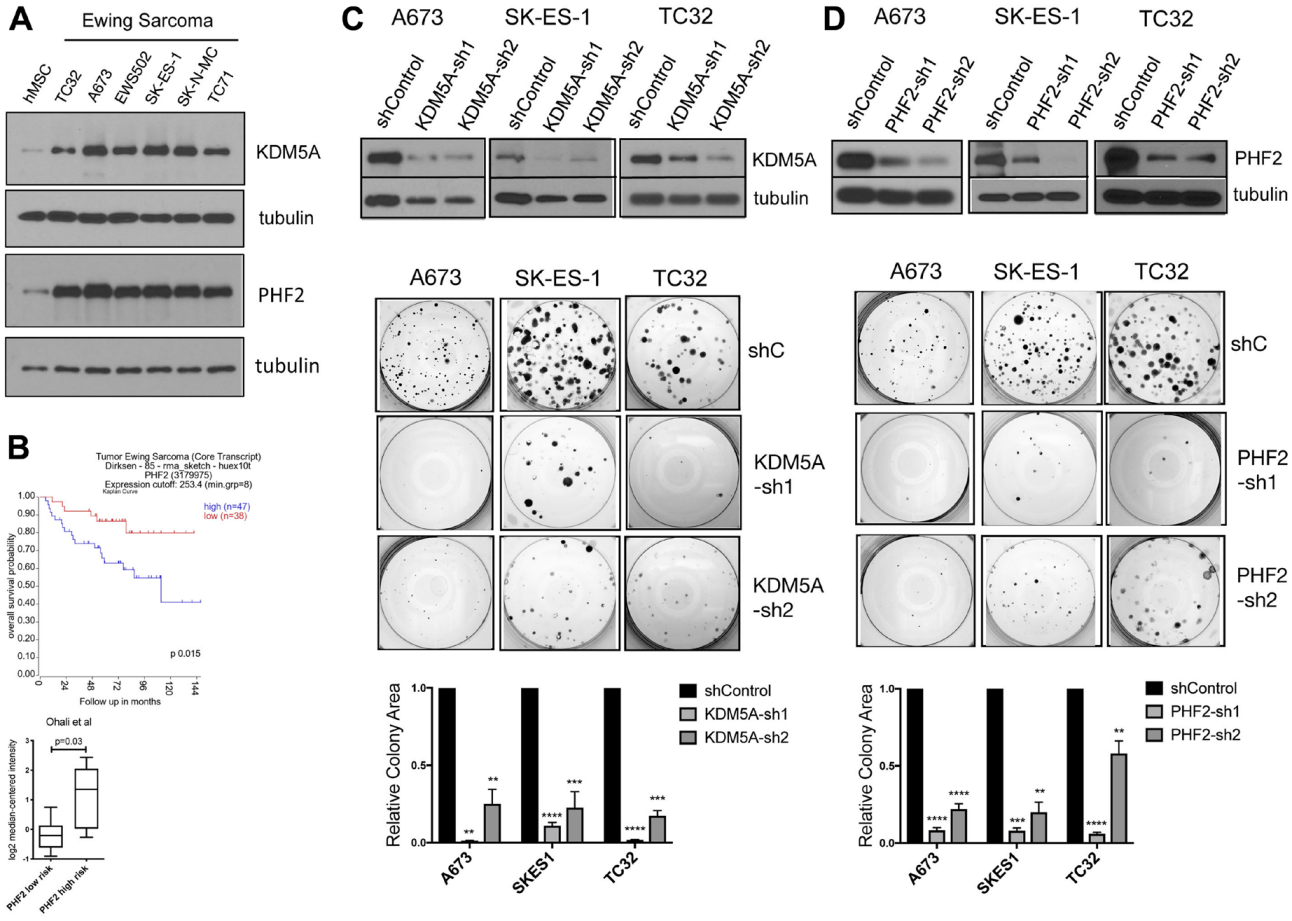
(**A**) Expression of KDM5A (top) and PHF2 (bottom) in hMSCs and patient-derived Ewing sarcoma cell lines, with tubulin as loading control. (**B**) Top panel: Kaplan-Meier analysis of Ewing sarcoma patient survival as a function of high versus low PHF2 expression in tumors (data from [46], visualized in R2, Genomics Analysis and Visualization Platform, https://hgserver1.amc.nl). Bottom panel: PHF2 expression in Ewing sarcoma tumors from patients with low- versus high-risk disease (data from [47], via Oncomine (https://www.oncomine.com)). Stable depletion of KDM5A (**C**) and PHF2 (**D**) inhibits colony growth in Ewing sarcoma cells. Top: KDM5A and PHF2 protein levels as a function of expression of control shRNA and 2 different targeting shRNAs, in the indicated cell lines, as determined by immunoblotting with tubulin as loading control. Center: Colony formation in a clonogenic assay from representative experiments. Bottom: Quantitative analysis of colony formation from 2–3 independent experiments for each manipulation and cell line, with each experiment performed in triplicate. Data plotted as mean and standard error; ^****^ *p* = 0.0001, ^***^ *p* < 0.001, ^**^ *p* < 0.01, relative to shControl, one-way ANOVA with multiple comparisons.

In previous studies, our group demonstrated disease-promoting properties for the Jumonji-domain histone demethylase (JHDM) KDM3A in Ewing sarcoma [19–21], as well as pre-clinical efficacy of a pan-JHDM inhibitor in this disease [22]. Seeking to further understand the biology of JHDMs in Ewing sarcoma, we noted the JHDMs KDM5A (JARID1A/RBBP2) and PHF2 (KDM7C/JHDM1E) to be consistently upregulated in expression in patient-derived Ewing sarcoma cell lines, relative to mesenchymal stem cells, the putative disease cell of origin (Figure 1A). Further, examination of public, outcome-annotated, patient tumor gene expression data showed higher PHF2 expression levels to significantly associate with more aggressive Ewing sarcoma disease (Figure 1B). Together, these data suggested that KDM5A and PHF2 could be novel disease-promoting factors in Ewing sarcoma.

**Figure 2:**
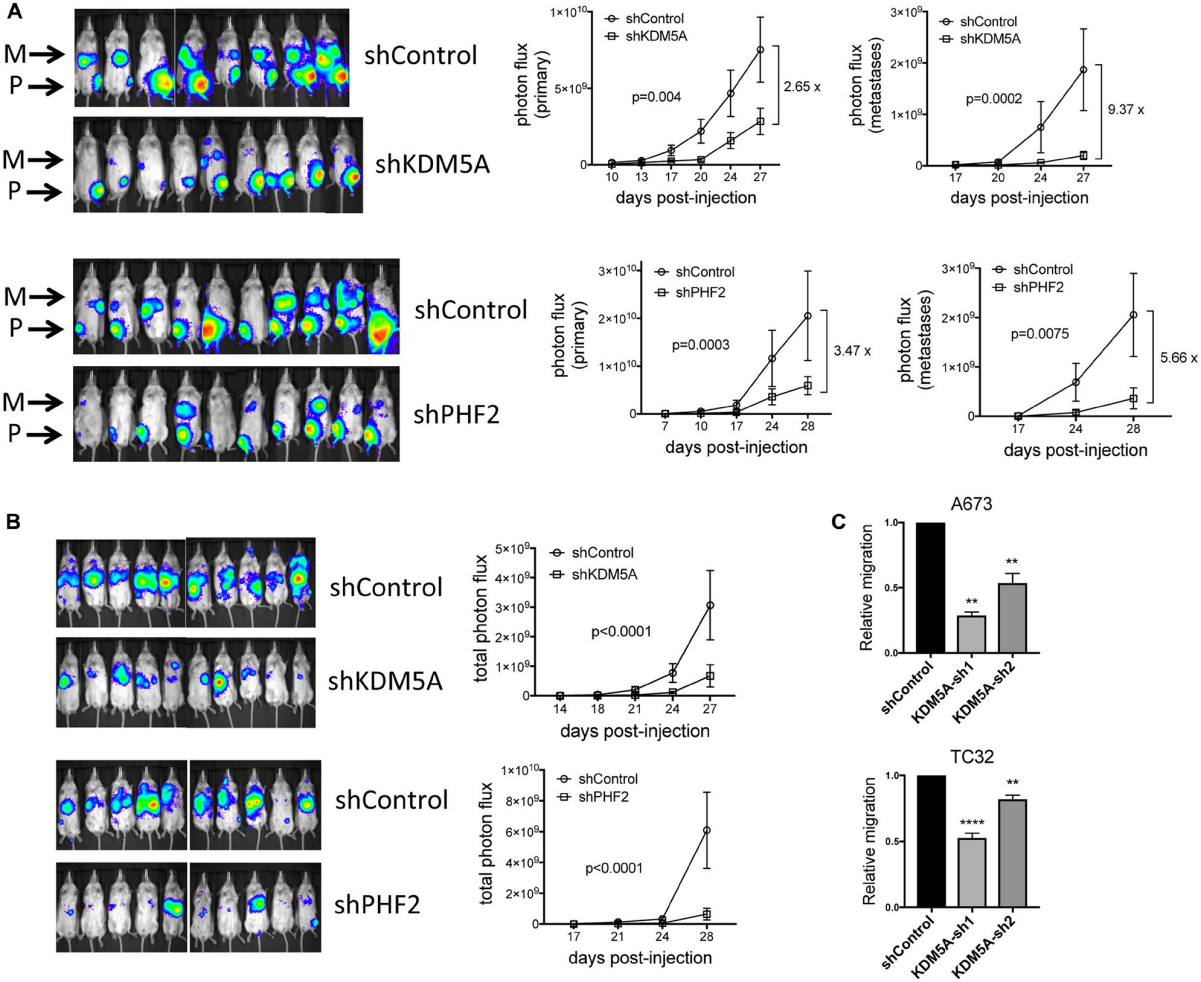
(**A**) Stable depletion of KDM5A and PHF2 inhibits tumorigenesis and metastasis in an orthotopic tibial injection model of Ewing sarcoma (A673 cells). Left: IVIS images of primary (P; tibia) tumor and metastasis (M) bioluminescence at end of study. Right: Quantitation of photon flux data from primary tumors and metastases (mean and standard error); *p*-values from two-way ANOVA with repeated measures; for KDM5A experiment, *n* = 8 and 9, respectively, for shControl and shKDM5A; for PHF2 experiment, *n* = 10 each for shControl and shPHF2. (**B**) Stable depletion of KDM5A and PHF2 inhibits metastasis in a tail vein injection model of Ewing sarcoma (A673 cells). Left: IVIS images of metastasis bioluminescence at end of study. Right: Quantitation of photon flux data (mean and standard error); *p*-values from two-way ANOVA with repeated measures; for KDM5A experiment, *n* = 10 each for shControl and shKDM5A; for PHF2 experiment, *n* = 10 each for shControl and shPHF2. (**C**) Stable depletion of KDM5A inhibits cell migration in a transwell assay (mean and standard error from 2 (A673 cells) and 3 (TC32 cells) independent experiments, each performed in triplicate; ^****^ *p* = 0.001, ^**^ *p* < 0.01, relative to shControl, one-way ANOVA with multiple comparisons).

To probe the functional roles of KDM5A and PHF2 in Ewing sarcoma, we stably depleted each factor in patient-derived Ewing sarcoma cell lines, using lentivirally delivered shRNAs (Figure 1C and 1D). Stable depletion of KDM5A and PHF2 each resulted in inhibition of colony formation (Figure 1C and 1D, and Supplementary Figure 1), indicating that both factors exert growth-promoting effects in Ewing sarcoma. To evaluate the role of KDM5A and PHF2 *in vivo*, we employed an orthotopic xenograft model of the disease. In this model, tumor cells stably expressing a luciferase reporter are injected into the proximal tibia, which results in the formation of both primary tumors at the injection site, and spontaneous metastatic disease. Compared to control shRNA animals, animals injected with KDM5A-depleted cells showed lower overall disease burden, and a reduction in primary tumor size (Figure 2A, top). Strikingly, animals with KDM5A-depleted cells showed a dramatic reduction in metastatic disease burden (Figure 2A, top). Similarly, depletion of PHF2 resulted in a reduction in overall disease burden and primary tumor size, and a more pronounced decrease in metastatic disease burden (Figure 2A, bottom). To verify that KDM5A and PHF2 depletion indeed inhibits metastasis, we employed a tail vein experimental metastasis assay. Similar to our findings in the orthotopic model, stable depletion of KDM5A and PHF2 each resulted in a robust decrease in metastatic disease burden in this model (Figure 2B). To evaluate whether modulation of cell motility could be contributing to the inhibition of metastasis, we examined the effects of factor depletion on cell migration in a transwell assay. We observed a decrease in cell migration upon depletion of KDM5A (Figure 2C). Together, these data indicate that KDM5A and PHF2 each promote growth and metastatic properties in Ewing sarcoma. Further, the data suggest that promotion of metastasis by KDM5A could be partly due to effects on cell motility.

### KDM5A and PHF2 promote pro-metastatic gene expression, and oppose repression of pro-metastatic genes by EWS/Fli1

**Figure 3:**
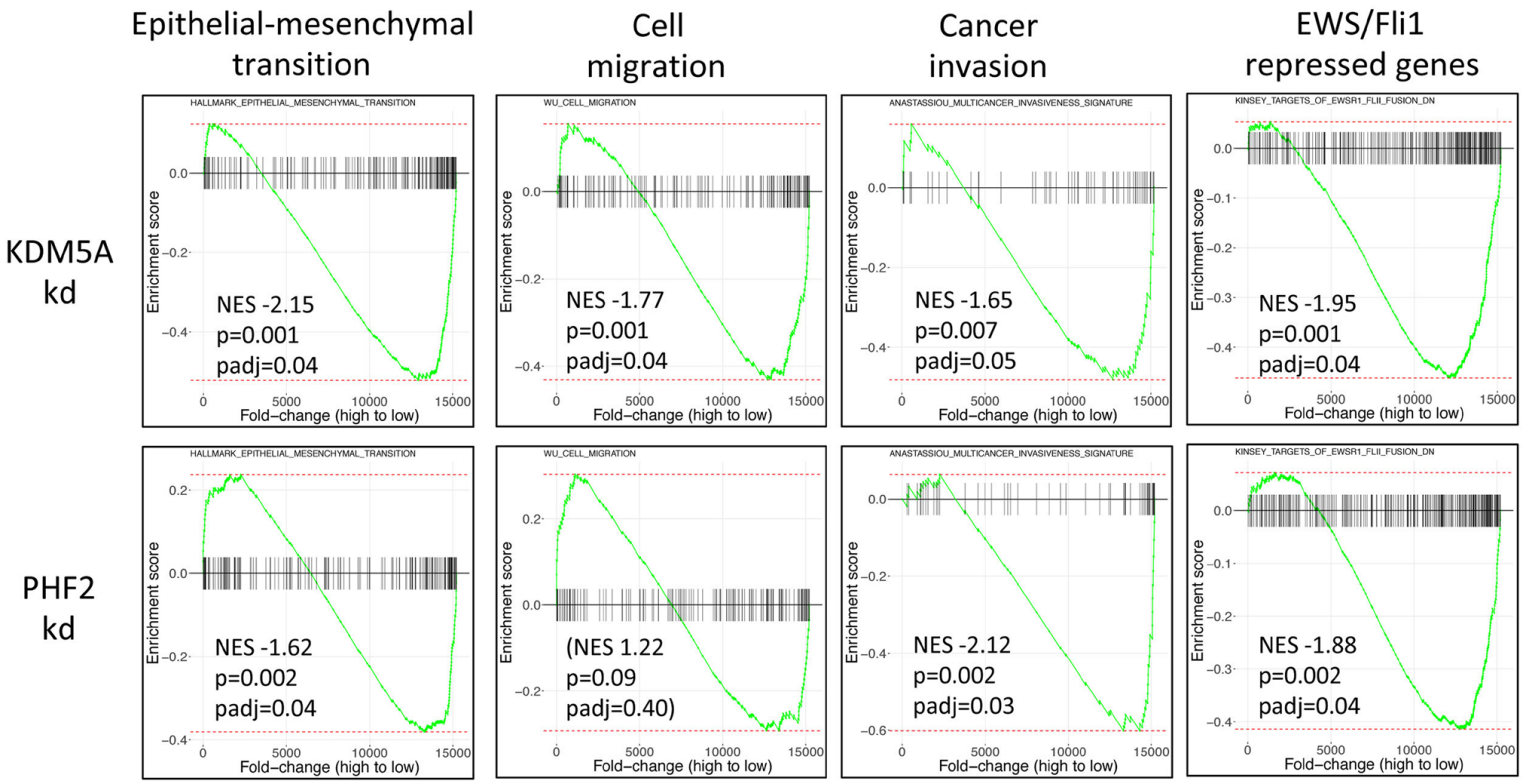
Gene Set Enrichment Analysis (GSEA) of KDM5A and PHF2 transcriptome data from A673 cells. Genes are ordered by fold-change in KDM5A (top) and PHF2 (bottom) knockdown cells compared to non-targeting shRNA controls. NES: normalized enrichment score; negative NES value indicates that gene set is predominantly downregulated knockdown cells; cell migration was not significantly enriched in the PHF2-regulated transcriptome.

To obtain further insight into mechanisms by which KDM5A and PHF2 function as disease promoting factors in Ewing sarcoma, we defined their respective transcriptomes using RNAseq, comparing gene expression in A673 Ewing sarcoma cells stably depleted of each factor and shRNA control cells (Supplementary Table 2). Strikingly, and in keeping with the metastasis-inhibiting phenotypic effects observed in our functional assays, Gene Set Enrichment Analysis (GSEA) revealed overall downregulation of genes belonging to the epithelial-mesenchymal transition signature, with depletion of KDM5A exerting a stronger effect than PHF2 depletion (Figure 3). KDM5A depletion, but not PHF2 depletion, also resulted in overall downregulation of cell motility genes (Figure 3). Further, KDM5A and PHF2 depletion each resulted in downregulation of a multicancer invasiveness signature; interestingly, PHF2 depletion exerted a stronger effect than KDM5A on this gene group (Figure 3). Taken together, these data indicate that KDM5A and PHF2 both positively control the expression of many genes implicated in metastasis promotion.

**Figure 4:**
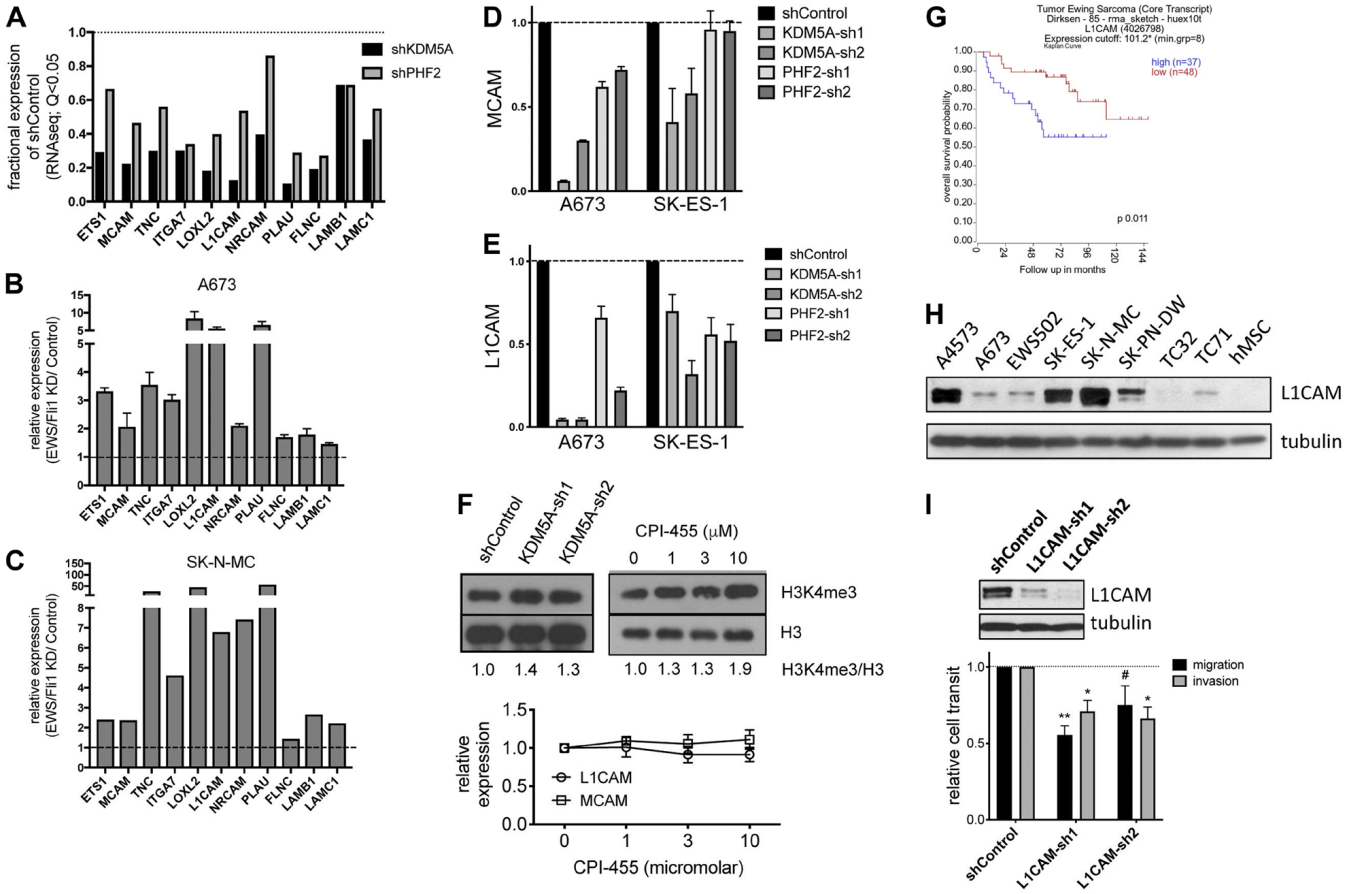
(**A**) Selected genes downregulated upon KDM5A and PHF2 depletion relative to shControl (RNAseq data). (**B**) Expression levels of same genes as in “A” in EWS/Fli1-depleted cells relative to control (A673-shA1c cells, −/+ Dox × 72 h; qRT-PCR). (**C**) Expression levels of same genes as in “A” in EWS/Fli1-depleted SK-N-MC cells relative to control (RNAseq data from [7]). (**D**) MCAM and (**E**) L1CAM expression levels in control, KDM5A-depleted and PHF2-depleted A673 and SK-ES-1 cells (qRT-PCR, 2 days following initial selection). (**F**) Top: H3K4me3 levels in control, KDM5A shRNA-depleted (2 days following initial selection), and CPI-455 treated (48 hours) A673 cells. Bottom: MCAM and L1CAM expression levels in control and CPI-455 treated cells (qRT-PCR at 48 hours; mean and standard deviation of expression in A673 and TC32 cells). (**G**) Kaplan-Meier analysis of Ewing sarcoma patient survival as a function of high versus low L1CAM expression in tumors (data from [46], via R2, Genomics Analysis and Visualization Platform, https://hgserver1.amc.nl). (**H**) Expression of L1CAM in patient-derived Ewing sarcoma cell lines and hMSC, with tubulin as loading control. (**I**) Stable shRNA-mediated depletion of L1CAM in A4573 cells inhibits cell migration and invasion (transwell assay without (migration) and with Matrigel (invasion); mean and standard error from 3 independent experiments, each performed in duplicate; ^**^ *p* < 0.01, ^*^ *p* < 0.05, ^#^ *p* = 0.056, relative to shControl, one-way ANOVA with multiple comparisons).

Another striking finding upon GSEA analysis of the transcriptome data was that KDM5A and PHF2 depletion each resulted in downregulation of the EWS/Fli1 repressed gene signature (Figure 3). As noted earlier (Introduction), EWS/Fli1 has recently been shown to exert inhibitory effects on: cell adhesion, motility and invasion; post-intravasation metastasis; expression of many pro-metastatic genes [10, 11, 13]. This observation raised the interesting possibility that the metastasis-promoting effects of KDM5A and PHF2, and the metastasis-dampening effects of EWS/Fli1, might act via opposing effects on shared downstream pathways. Inspection of individual genes of interest revealed that KDM5A and PHF2 each positively control the expression of Ets1 and MCAM (Figure 4A), genes belonging to a metastasis-promoting pathway recently identified in Ewing sarcoma by our group [21], and negatively regulated by EWS/Fli1 (Figure 4B and 4C). Similarly, we observed the same regulatory effects on another recently demonstrated pro-metastatic gene in Ewing sarcoma, TNC [23] (Figure 4A–4C). Further examination of genes subject to positive regulatory control of KDM5A and PHF2, and negative regulatory control by EWS/Fli1, revealed a number of additional genes not previously investigated in Ewing sarcoma, but shown to promote metastatic properties in other cancers, including ITGA7, LOXL2, L1CAM, NRCAM, PLAU, FLNC, LAMB1 and LAMC1 (Figure 4A–4C).

Examination of regulatory control of MCAM in different Ewing sarcoma cell lines revealed positive regulation by KDM5A in both A673 and SK-ES-1 cells, and by PHF2 in A673 cells (Figure 4D). L1CAM, another cell surface protein strongly implicated in cancer progression and metastasis [24, 25], demonstrated positive regulatory control by KDM5A and PHF2 in both cell lines (Figure 4E). KDM5A uses its demethylase activity, directed at H3K4me2/3 activating histone marks, to repress gene expression, but has also been shown to be able to activate gene expression via less well defined mechanisms (as recently reviewed [26]). In principle, KDM5A could also utilize its demethylase activity to activate gene expression via repression of a downstream repressor. To determine whether KDM5A demethylase activity contributes to positive regulation of MCAM and L1CAM expression, we employed the KDM5-specific inhibitor CPI-455 [27]. Treatment of Ewing sarcoma A673 cells with CPI-455 resulted in increased levels of H3K4me3, similar to KDM5A depletion (Figure 4F). However, in contrast to KDM5A depletion, CPI-455 treatment did not affect expression levels of MCAM or L1CAM. These results suggest that KDM5A positively controls MCAM and L1CAM expression via a demethylase-independent mechanism.

Genes expressed on the cell surface present potential therapeutic targets for antibodies, small molecules, or, more recently, immune (CAR T-cell) based approaches. We have previously identified MCAM as one such druggable target in Ewing sarcoma [21]. L1CAM presents another such candidate. Similar to PHF2, higher L1CAM expression is significantly associated with inferior clinical outcome in Ewing sarcoma (Figure 4G). L1CAM is also an adverse prognostic factor in Rhabdomyosarcoma [28], the most common pediatric soft tissue sarcoma. L1CAM is additionally highly expressed in Neuroblastoma, the most common extracranial solid malignant neoplasm of childhood, where it is being targeted via CAR T-cell approaches [29]. Our expression survey revealed a broad range of L1CAM expression levels in patient-derived Ewing sarcoma cell lines, ranging from low levels in TC32 cells to high levels in A4573, SK-ES-1 and SK-N-MC cells (Figure 4H). Furthermore, depletion of L1CAM in A4573 cells, which express high L1CAM levels and also manifest robust motile/invasive properties *in vitro*, resulted in inhibition of migration and invasion in transwell assays (Figure 4I). Thus, L1CAM is an adverse prognostic factor in Ewing sarcoma, and promotes migratory and invasive properties.

### KDM5A and PHF2 inhibit disease-promoting properties in more metastatically potent EWS/Fli1^low^ cells

**Figure 5:**
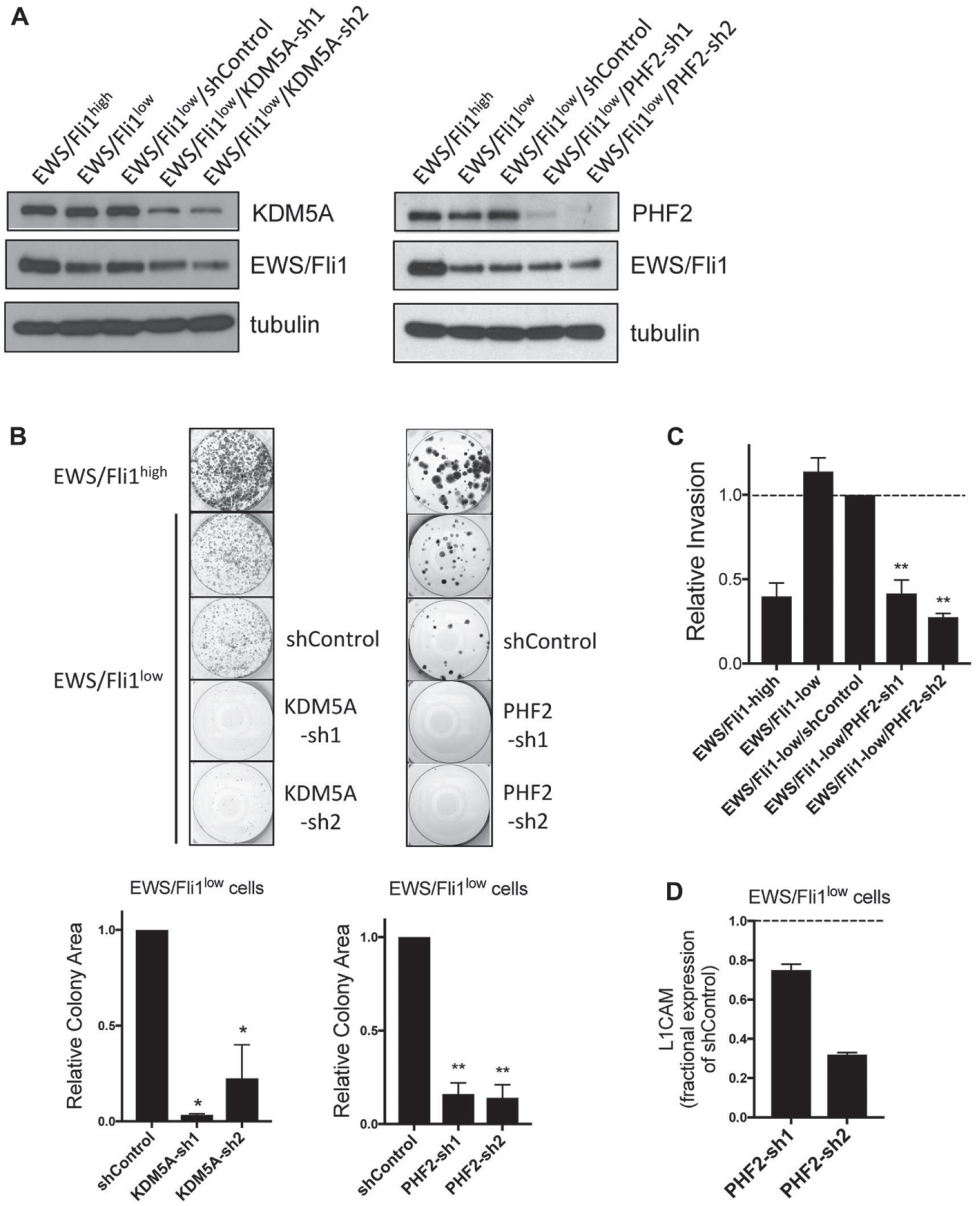
(**A**) KDM5A (left) and PHF2 (right) depletion in EWS/Fli1^low^ cells. Immunoblot data with tubulin as loading control. (**B**) Colony formation in clonogenic assay, shown as images from representative experiment (top), and quantifications from 2 independent experiments, each performed in triplicate (bottom); ^*^ *p* < 0.05, ^**^ *p* < 0.01, relative to EWS/Fli1^low^/shControl, one-way ANOVA with multiple comparisons. (**C**) Transwell invasion by the indicated cells. Data from 2 independent experiments, each performed in triplicate; mean and standard error; data from EWS/Fli1^low^/shControl cells set to 1; ^**^ *p* < 0.01, relative to EWS/Fli1^low^/shControl, one-way ANOVA with multiple comparisons. (**D**) Fractional expression of L1CAM in PHF2-depleted EWS/Fli1^low^ cells, relative to EWS/Fli1^low^/shControl cells; mean and standard error of RNA levels from 2 independent experiments, each performed in triplicate.

Having shown that KDM5A and PHF2 promote metastatic properties in Ewing sarcoma, and exert effects on metastasis in opposition to those of EWS/Fli1, we wondered whether inhibition of KDM5A or/and PHF2 could impair the biological properties of more metastatically potent EWS/Fli1^low^ cells [10, 11, 13]. To answer this question, we turned to the well-characterized Ewing sarcoma A673 cell line with inducible EWS/Fli1 shRNA-mediated depletion [11, 30]. We first verified that EWS/Fli1^low^ cells maintain high KDM5A and PHF2 expression (Figure 5A). We next stably depleted KDM5A and PHF2 expression in EWS/Fli1^low^ cells (Figure 5A), and examined the effects on growth and invasive properties of this cell population. Consistent with previously published findings [11], EWS/Fli1^low^ cells grew more slowly, as determined by a clonogenic colony formation assay (Figure 5B). Depletion of KDM5A and PHF2 in EWS/Fli1^low^ cells each resulted in further, and substantial, inhibition of colony growth in a clonogenic assay (Figure 5B). While inhibited in growth, EWS/Fli1^low^ cells show substantially augmented invasive potency, as previously published [10, 11], and verified in our invasion assay (Figure 5C). Strikingly, stable depletion of PHF2 resulted in potent inhibition of invasion by EWS/Fli1^low^ cells; in fact, PHF2 depletion effectively reversed the augmented invasive ability of this cell population (Figure 5C). We further found that PHF2 retained positive regulatory control of L1CAM in EWS/Fli1^low^ cells (Figure 5D). Taken together, our findings indicate that KDM5A and PHF2 retain their growth-promoting properties in EWS/Fli1^low^ cells, and that PHF2 contributes to the increased invasive potency of this cell population.

### KDM5A and PHF2 inhibit metastasis of EWS/Fli1^low^ cells

**Figure 6:**
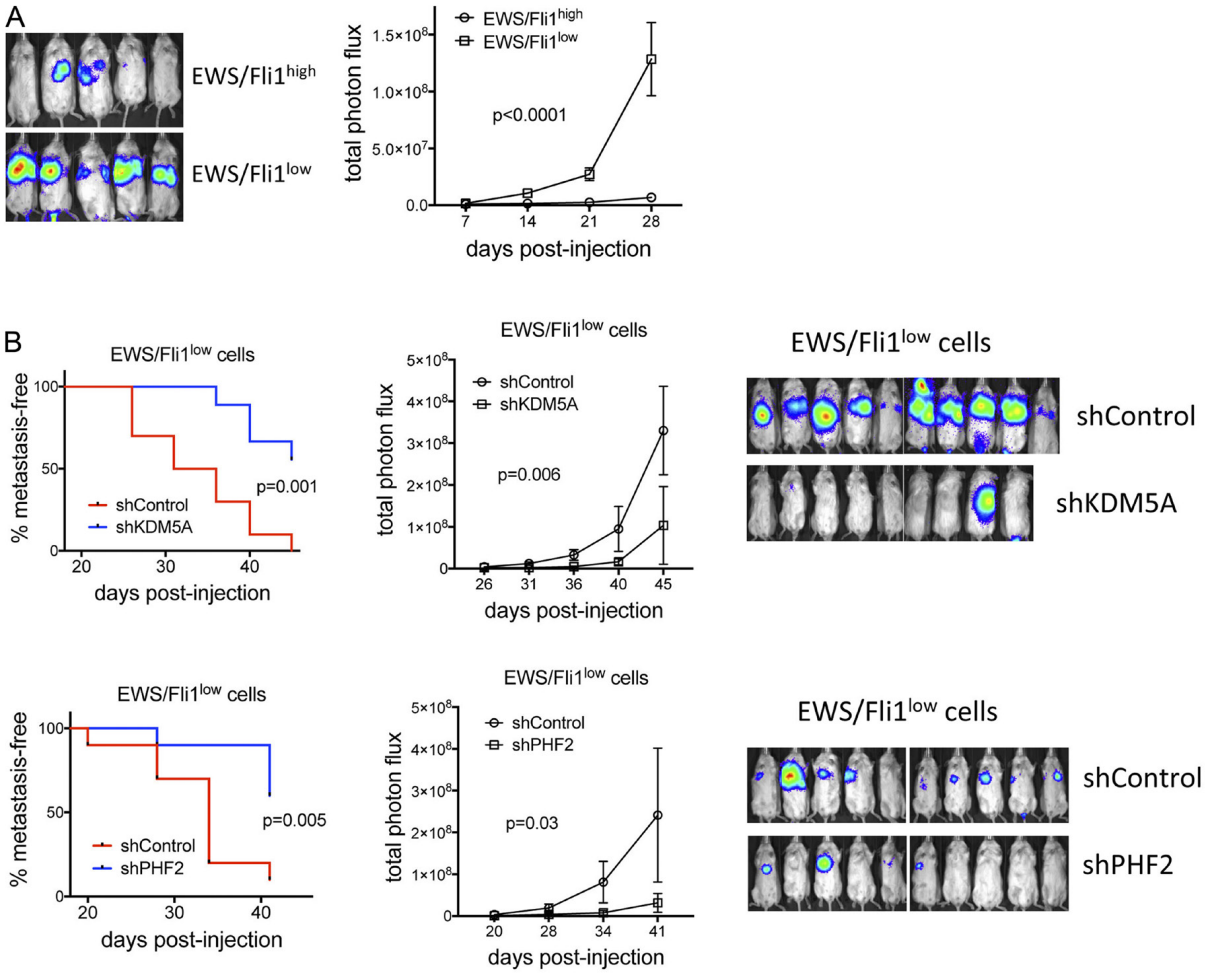
(**A**) Following the experimental protocol of Franzetti et al. [11] A673-shA1c cells −/+ Dox (EWS/Fli1^high^/EWS/Fli1^low^) were injected into the tail vein, and metastases were allowed to form in the absence of further Dox treatment (to allow recovery of cell growth in EWS/Fli1^low^ cells). IVIS data at end of study (left) and quantification of photon flux (right); *n* = 5 for each group; mean and standard error, *p*-values from two-way ANOVA with repeated measures. (**B**) Effects of KDM5A (top) and PHF2 (bottom) depletion on metastasis of EWS/Fli1^low^ cells, using the same experimental approach and analysis as in “A”; for KDM5A experiment, *n* = 10 and 9, respectively, for shControl and shKDM5A; for PHF2 experiment, *n* = 10 each for shControl and shPHF2. “Metastasis-free” animals were defined as those with total photon flux < 1 × 10^7^.

Given the above findings, we next asked whether depletion of KDM5A or/and PHF2 inhibits the metastatic potency of EWS/Fli1^low^ cells. We first verified the greater metastatic potency of EWS/Fli1^low^ cells, relative to EWS/Fli1^high^ cells, in our tail vein injection xenograft model in NOD/SCID-Gamma mice (Figure 6A), using the experimental approach previously described [11]. Next, we compared metastatic potency of KDM5A-depleted and PHF2-depleted EWS/Fli1^low^ cells, to EWS/Fli1^low^/shControl cells, in the same model system. KDM5A depletion and PHF2 depletion each resulted in significantly lower metastatic burden relative to control cells (Figure 6B). Thus KDM5A and PHF2 depletion both attenuate the metastatic potency of EWS/Fli1^low^ cells.

## DISCUSSION

Ewing sarcoma is an aggressive cancer with a strong propensity for metastasis. However, the mechanistic basis for this metastatic propensity remains poorly understood. Moreover, recent studies have surprisingly revealed that EWS/Fli1, the driver oncofusion in this disease, inhibits, rather than promotes, many important metastatic properties in Ewing sarcoma [10, 11, 13]. Recent investigations have also underscored the prominent role that epigenetic mechanisms play in Ewing sarcoma pathogenesis [7, 31, 32]. However, detailed understanding of these mechanisms, including potential roles in metastasis and other aspects of disease progression, remains to be attained. In the present study, we present evidence that epigenetic mechanisms, involving the chromatin factors KDM5A and PHF2, importantly contribute to Ewing sarcoma progression, in part through their opposing effects on EWS/Fli1-controlled gene expression.

KDM5A has recently been found to be upregulated in expression and disease-promoting in a number of other cancers, through both pro-growth and pro-metastatic effects (as recently reviewed [26]). KDM5A is a multidomain protein, capable of exerting both activating and repressive effects on gene expression in normal and cancer cells [26]. The demethylase activity of KDM5A (directed at activating H3K4 histone methyl marks) plays a role in its repressive activity, and, interestingly, may also contribute to gene activation, through unknown mechanisms [33, 34]. KDM5A can also activate gene expression through demethylase-independent mechanisms, also largely uncharacterized [35]. Our studies provide evidence that, in Ewing sarcoma, KDM5A activates expression of L1CAM (and MCAM) via a demethylase-independent mechanism. This is similar to investigations of the pro-metastatic role of KDM5A in breast cancer, where KDM5A was found to upregulate expression of TNC (also identified as a KDM5A-dependent gene in our studies) via a demethylase-independent mechanism [35]. PHF2 has been less extensively studied in cancer (as recently reviewed [26]). Interestingly, investigations in adult cancers of epithelial origin have so far provided evidence for disease-suppressive roles [36, 37]. Our studies thus provide the first evidence for a disease-promotional role for PHF2 in cancer, and highlight the context-dependent action of epigenetic regulators in biology. PHF2 demethylase activity, directed at repressive H3K9 and H3K27 histone methyl marks, is likely to be responsible for PHF2-dependent gene expression in Ewing sarcoma, although this remains to be determined. Interestingly, similar to our studies, PHF2 has recently been found to function as a pro-proliferative factor in neural progenitors [38].

**Figure 7:**
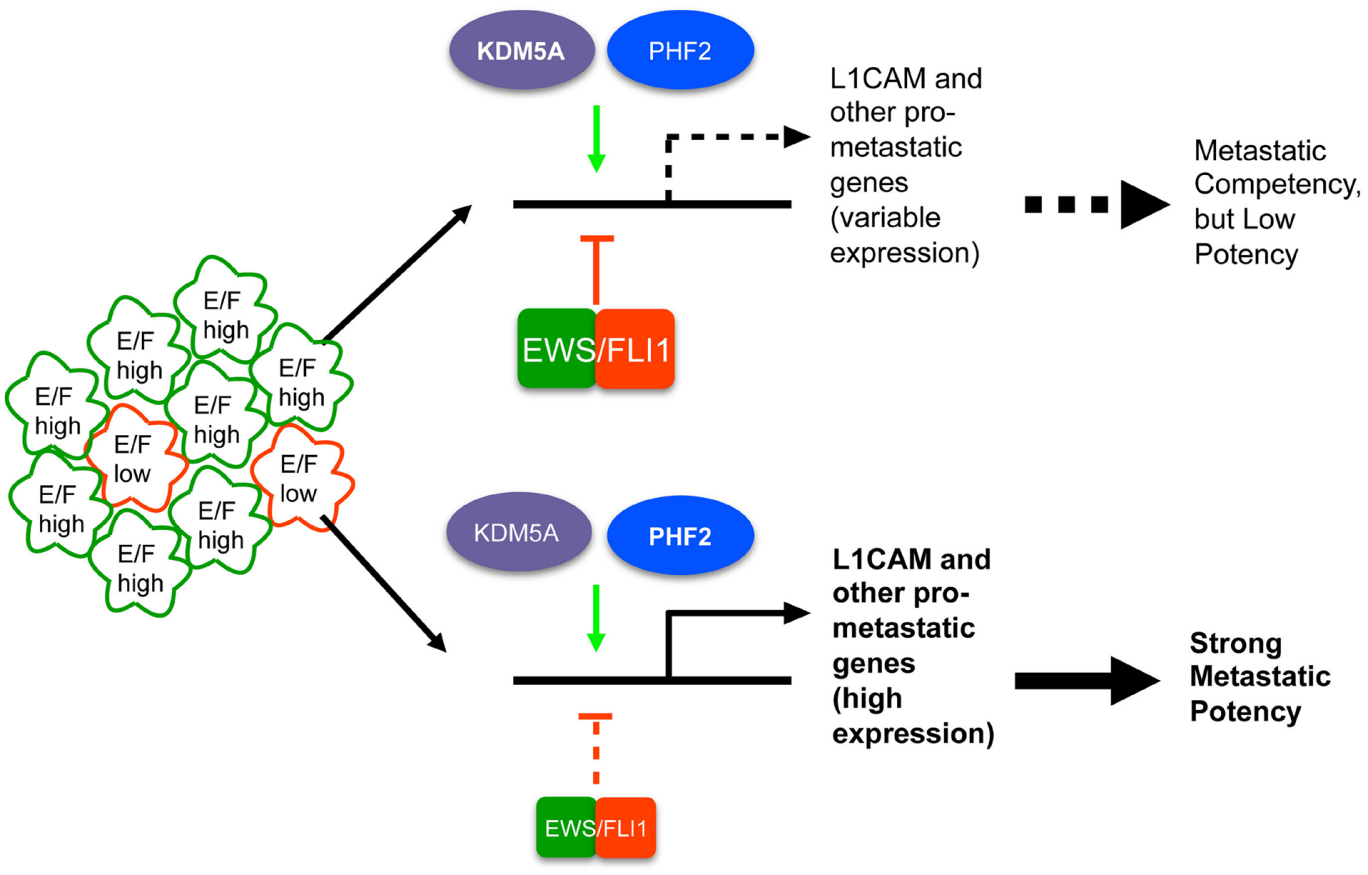
Proposed model of KDM5A and PHF2 action in Ewing sarcoma based on our findings. In EWS/Fli1^high^ cells, which constitute the majority of Ewing sarcoma cell/tumor populations, KDM5A and PHF2 positively control expression of L1CAM and other metastasis-promoting genes, in opposition to the repressive effects of EWS/Fli1; we propose that such mechanisms help maintain metastatic competency in the context of high EWS/Fli1 expression. KDM5A, and especially PHF2, also exert strong disease-promoting effects in EWS/Fli1^low^ cells, thus contributing to the enhanced metastatic potency of this cell population (see manuscript text for further discussion).

Our studies identify striking opposition of the effects of KDM5A/PHF2 and EWS/Fli1 on the expression of motility/invasion/metastasis-associated genes, and corresponding phenotypic effects on cell motile/invasive properties and post-intravasation metastasis. Our findings in “bulk” Ewing sarcoma cell populations, known to be composed predominantly of cells with high EWS/Fli1 expression [11], suggest that KDM5A and PHF2 may play an important role in helping restrain the metastasis-repressive effects of EWS/Fli1, and as such to help maintain metastatic competency in Ewing sarcoma. Importantly, our studies further show that KDM5A and PHF2 retain disease-promoting properties in the more metastatically potent EWS/Fli1^low^ cells. In fact, PHF2 not only retains its growth promoting effects, but strongly contributes to the enhanced invasive potency of this important cell population. Equally importantly, our *in vivo* model studies show that KDM5A and PHF2 depletion is each able to attenuate the metastatic potency of EWS/Fli1^low^ cells. Together, our studies thus identify specific, and potentially targetable, epigenetic mechanisms that promote metastatic properties both in the context of “bulk” (predominantly EWS/Fli1^high^) cell populations, and specifically in more metastatically potent EWS/Fli1^low^ cells, as summarized in the diagram in Figure 7.

Our studies further identify a group of genes previously implicated in metastasis, whose opposing regulatory control by KDM5A/PHF2 and EWS/Fli1 may importantly contribute to modulation of Ewing sarcoma metastatic potency. This gene group includes MCAM and TNC, both recently established metastasis-promoting factors in Ewing sarcoma [21, 23]. Such genes/proteins, upregulated in expression in EWS/Fli1^low^ cells, may provide an alternative means of inhibiting the aggressive biology of this cell population. Our studies suggest that L1CAM may be another such gene/protein. L1CAM is overexpressed in a variety of cancers, and has been shown to be capable of exerting both tumor and metastasis-promoting effects [24, 25]. Interestingly, L1CAM has previously been identified as part of a high-risk gene signature in the pediatric cancer Rhabdomyosarcoma [28]. Here, we identify L1CAM as variably expressed in Ewing sarcoma, strongly induced upon EWS/Fli1 downregulation, and strongly associated in expression with adverse clinical outcome. We further show that L1CAM promotes Ewing sarcoma cell migratory and invasive properties when expressed at high levels. L1CAM is also highly expressed in the pediatric cancer Neuroblastoma where it is being targeted via CAR T cell-based approaches [29]. It will be of interest to determine whether similar approaches could be an efficacious means to attenuate Ewing sarcoma metastatic potency. It will also be of interest to more fully understand mechanisms controlling its highly variable expression in Ewing sarcoma, which could present alternative opportunities to inhibition of L1CAM effects.

Taken together, our studies uncover the existence of epigenetic mechanisms in Ewing sarcoma whose gene regulatory effects intersect those of EWS/Fli1 so as to promote disease properties in cells with high as well as low EWS/Fli1 expression. Further understanding of such mechanisms should help illuminate the molecular basis of metastatic propensity in Ewing sarcoma, as well as present new approaches to metastasis inhibition, including attenuation of the high metastatic potency of cells with low EWS/Fli1 expression.

## MATERIALS AND METHODS

### Cell lines and culture conditions

Ewing sarcoma patient-derived cell lines, human mesenchymal stem cells (Lonza), and their respective culture conditions have been previously described [20, 21, 39]. The A673-shA1c cell line with Doxycycline-inducible EWS/Fli1 shRNA expression was kindly provided by Dr. Olivier Delattre, and cultured as described [30]. All cell lines were authenticated at our institution by STR profiling and repeatedly verified to be mycoplasma-free.

### Stable silencing of gene expression

shRNA-mediated gene expression silencing via lentiviral delivery was performed as previously described [21]. The control, non-targeting shRNA consisted of a scrambled sequence (Addgene plasmid 1864; [40]). ShRNAs 1 and 2 for KDM5A correspond to TRCN0000014632 and TRCN0000329872, respectively; shRNAs 1 and 2 for PHF2 correspond to TRCN0000019238 and TRCN0000230571, respectively; shRNAs 1 and 2 for L1CAM correspond to TRCN0000299624 and TRCN0000303668, respectively (all Sigma Mission shRNAs, distributed via the University of Colorado Cancer Center Functional Genomics Core Facility). Following lentiviral transduction, cells were selected with puromycin (2 μg/ml for A673, TC32 and A673-shA1C cells; 1 μg/ml for SK-ES-1 and A4573 cells); A673-shA1c cells were treated with Doxycycline (Dox; 2 μg/ml) beginning 48 hours prior to transduction.

### RNA expression analysis

Cells were harvested at 70–80% confluence in TRIzol (Invitrogen), and RNA was extracted per manufacturer’s instructions. RNA levels of specific transcripts were assessed by qRT-PCR (using qScript SuperMix, PerfeCTa SYBRgreen, Quantabio) with 18S RNA as the internal control (primers are listed in Supplementary Table 1).

### Protein expression analysis

Protein expression levels in whole cell extracts were determined as previously described [41]. Primary antibodies used were: Fli1 (Abcam, ab15289, 1:1000); KDM5A (Abcam, ab70892, 1:500); PHF2 (Cell Signaling Technology, #3497, 1:1000); L1CAM (Cell Signaling Technology, #89861S, 1:1000) and α-tubulin (Sigma, #T5168, 1:20,000). For histone mark analysis, harvested cells were resuspended in Triton Extraction Buffer (TEB: PBS containing 0.5% Triton X-100, 2 mM phenylmethylsulfonyl fluoride (PMSF), and 0.02% (w/v) NaN_3_) at a density of 10^7^ cells/ml and lysed for 10 minutes on ice. The resulting suspension was spun 10 minutes at max speed at 4°C to pellet nuclei. Nuclei were washed in one half of the original volume of TEB, collected by centrifugation as before, resuspended in 0.2 N HCl at a density of 4 × 10^7^ nuclei/ml, and rotated overnight at 4°C to extract histones. Extracted histones were collected by spinning 10 minutes at max speed at 4°C, and collecting the supernatant. Histone extracts were subjected to SDS-PAGE and immunoblotting as previously described [20]. Primary antibodies used were: H3K4me3 (Cell Signaling Technologies, #9751, 1:1000) and H3 (Abcam, #1791,1:1000).

### Global gene expression analysis

Biological triplicates of A673 cells stably transduced with Scrambled shRNA control, or shRNAs targeting KDM5A or PHF2, were harvested at 70–80% confluence. RNA was isolated using TRIzol extraction, and further purified using the Qiagen MinElute column kit. Samples were submitted to the University of Colorado Cancer Center Microarray and Genomics shared resource for analysis of RNA quality, library preparation, and directional mRNA next-generation sequencing at 50 cycles of single-end reads on an Illumina Hi-Seq 4000 instrument. Sequencing data were processed through a custom computational pipeline consisting of the open-source gSNAP, Cufflinks, and R for alignment and discovery of differential gene expression [42, 43]. Fragments per kilobase of exon per million mapped reads (FPKM) were used for comparison of transcript levels, and significant differences in gene expression were calculated using ANOVA in R. Gene Set Enrichment Analysis was performed using fgsea R package (https://www.biorxiv.org/content/10.1101/060012v1) and MSigDB gene sets and published signatures. Gene sets with *p* < 0.05 (after 1000 gene set permutations) were deemed to be enriched in each group. Gene expression profiling data have been deposited into the NCBI Gene Expression Omnibus database (accession number GSE156387).

### Colony formation assays

Cells were stably transduced with Scrambled shRNA control, or KDM5A, PHF2 or L1CAM targeting shRNAs, and harvested at 70–80% confluence. For clonogenic assays, cells were plated at 500 cells per well in six-well plates. Colonies were stained 14 days later with 0.1% crystal violet in 25% MeOH, and quantified using Metamorph imaging software. Soft agar assays were performed as described [41], beginning with 3 × 10^4^ cells/well, and quantified using Metamorph imaging software.

### Cell migration and invasion assays

For transwell migration experiments, cells were washed with serum-free media, and 20,000 cells were plated in replicate in 200 μl of serum-free media in the top of the well insert (8 μm pore, BD Biosciences, #353097). Inserts were then placed in a 24-well companion plate with 600 μl of media containing 10% fetal bovine serum (FBS) as a chemoattractant. After incubation for 16 hours, cells were fixed for 20 minutes in 70% ethanol, and unmigrated cells were removed by cleaning the top of the membrane with a cotton swab. Migrated cells were permeabilized for 5 minutes in 0.3% Triton X-100 in PBS. Cells were stained with 3 μg/ml DAPI in PBS for 20 minutes. Five random fields at 10× power were taken of each well. Quantitation was carried out using the Nikon NLS software object count. Transwell invasion experiments were carried out in a similar manner using Matrigel Invasion Chambers (Corning, #354480) and incubation for 36 hours.

### Tail vein injection and tibial bone injection xenograft studies

Cells were transduced with a lentiviral GFP/luciferase dual reporter (SFG-NES-TGL [44]) and sorted for GFP expression using flow cytometry. GFP-positive cells were then transduced with either Scrambled control or targeting shRNA and puromycin-selected. For injections, cells were harvested, washed, and resuspended in serum-free/antibiotic-free medium. For tail vein injections, 5 × 10^6^ (A673) or 2 × 10^6^ (A673-shA1c) cells in 200 μl were injected into NOD/SCID Gamma (NSG) mice (bred on site in a specific pathogen-free facility), as previously described [21]. For tibial bone injections, 1 × 10^6^ cells in 40 μl were injected into the tibia of NSG mice, under inhalation anesthesia, as previously described [45]. All experiments used 8–10 animals per group, on average 3 months in age, male and female; control and experimental groups in each study were matched as closely as possible for age, sex and health status; group assignment was otherwise random; animals were housed in a specific pathogen-free facility. Tumor burden was tracked at regular intervals using bioluminescent imaging under inhalation anesthesia, as previously described [21]. All animal experiments were in compliance with ethical regulations as approved by the University of Colorado School of Medicine Institutional Animal Care and Use Committee (IACUC), and were conducted in an AAALAC (Association for Assessment and Accreditation of Laboratory Animal Care International) accredited animal facility.

## SUPPLEMENTARY MATERIALS




