# Supplementary material for: KDM5A and PHF2 positively control expression of pro-metastatic genes repressed by EWS/Fli1, and promote growth and metastatic properties in Ewing sarcoma
Source: Oncotarget. 2020 Oct 27;11(43):3818–31. doi: 10.18632/oncotarget.27737 (PMC7597412; doi:10.18632/oncotarget.27737)
Supplement: Supplementary file 1 [file oncotarget-11-3818-s001.pdf]

KDM5A and PHF2 positively control expression of pro-metastatic genes repressed by EWS/Fli1, and promote growth and metastatic properties in Ewing sarcoma

SUPPLEMENTARY MATERIALS

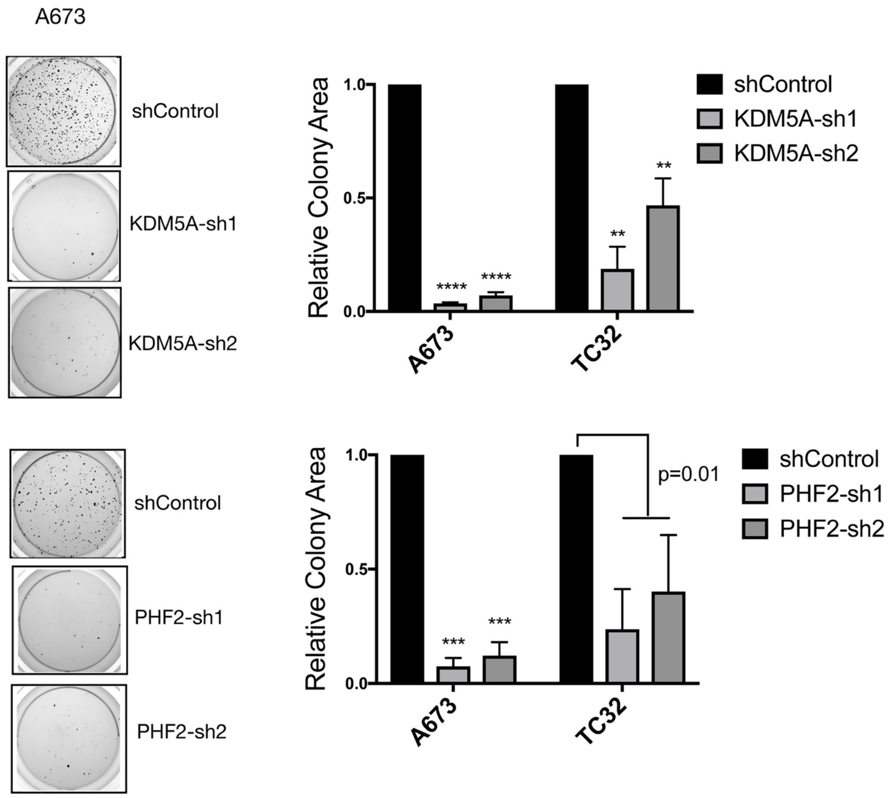

Supplementary Figure 1: Soft agar assay data from KDM5A and PHF2 depletion experiments in Figure 2. Data from representative experiment in A673 cells (left), and quantified data from 2 (A673 cells) and 3 (TC32 cells) independent experiments, each performed in triplicate (right); mean and standard error; $^{****}p = 0.0001$, $^{***}p < 0.001$, $^{**}p < 0.01$, relative to shControl, one-way ANOVA with multiple comparisons; $p = 0.01$, two-way student t-test with unequal variance.

Supplementary Table 1: Primers used

qRT-PCR	
Ets1 For	TCATTTCTTTGCTGCTTGGA
Ets1 Rev	CTCACCATCATCAAGACGGA
MCAM For	AGCTCCGCGTCTACAAAGC
MCAM Rev	CTACACAGGTAGCGACCTCC
TNC For	CCTAGGTCTCTCGCCCATC
TNC Rev	GGCTCTAGGGCTCTAGGGATT
ITGA7 For	CTGACTCCATGTTCGGGATCA
ITGA7 Rev	CACCTGTGAAGGTTTGGCG
LOXL2 For	CACTGCGGATCCCTGAAAC
LOXL2 Rev	CCTGTCTTCGGGCTGATG
L1CAM For	CCGACAACCACTCAGACTACA
L1CAM Rev	CCGGAGGTCAATGGGTTCC
NRCAM For	GAGGTGTCTAGCCCAGTGGA
NRCAM Rev	ATGCGGGAAACTTTGAAGAA
PLAU For	TGACCCACAGTGGAAAACAG
PLAU Rev	CCAGCTCACAATTCCAGTCA
FLNC For	CACATCAAGCTCGTGTCCAT
FLNC Rev	GGAGTAGTGCAGGATCAGCG
LAMB1 For	GAACTCTTCTGGGGAGACCC
LAMB1 Rev	CAACGCAGACACACTGGC
LAMC1 For	CAGTACCCCAGCTCCATCAA
LAMC1 Rev	GTAAATGGCAAAGCTCTCCG

Supplementary Table 2: Genes significantly changed in expression ($Q < 0.05$) upon KDM5A stable knockdown in A673 cells. See Supplementary Table 2
